# Subjective Symptoms of Male Workers Linked to Occupational Pesticide Exposure on Coffee Plantations in the Jarabacoa Region, Dominican Republic

**DOI:** 10.3390/ijerph15102099

**Published:** 2018-09-25

**Authors:** Hans-Peter Hutter, Michael Kundi, Kathrin Lemmerer, Michael Poteser, Lisbeth Weitensfelder, Peter Wallner, Hanns Moshammer

**Affiliations:** Department of Environmental Health, Center for Public Health, Medical University Vienna, 1090 Wien, Austria; hans-peter.hutter@meduniwien.ac.at (H.-P.H.); michael.kundi@meduniwien.ac.at (M.K.); kathrin.lemmerer@meduniwien.ac.at (K.L.); michael.poteser@meduniwien.ac.at (M.P.); peter.wallner4@gmail.com (P.W.); hanns.moshammer@meduniwien.ac.at (H.M.)

**Keywords:** coffee plantation, occupational health, pesticide, self-reported symptoms, sprayer

## Abstract

Acute and sub-acute effects of pesticide use in coffee farmers have rarely been investigated. In the present field study, self-reported health symptoms from 38 male pesticide users were compared to those of 33 organic farmers. Results of cytological findings have been reported in an accompanying paper in this issue. The present second part of the study comprises a questionnaire based survey for various, potentially pesticide related symptoms among the coffee farmers. Symptom rates were generally higher in exposed workers, reaching significance in nine out of 19 assessed symptoms. Significantly increased symptom frequencies were related to neurotoxicity, parasympathic effects and acetylcholine esterase inhibition, with the highest differences found for excessive salivation, dizziness and stomach ache. We revealed a lack of precautionary measures in the majority of farmers. Better education, regulations, and safety equipment are urgently needed.

## 1. Introduction

Exposure to pesticides poses one of the most important occupational risks to farmers in countries in the Global South (e.g., [1,2,3,4,5]), being associated with a wide range of symptoms and disorders including respiratory problems, metabolic disorders, neurodegenerative diseases or cancer (e.g., [6,7,8,9]) aside from several biomarker changes (e.g., [10,11,12]). Identification of the hazards of pesticide use and the establishment of safe methods of pesticide handling is a challenge for occupational health and safety (see [13] for regulations or [14] for safety training).

While China and the United States are the leading countries in pesticide use, occupational pesticide intoxications are frequently reported in farm workers of export oriented or agricultural mass products in the tropical zone, where high requirements on productivity and quality are challenged by climatically fostered abundance of fungi and other pests [15]. Unfortunately, these parts of the world hold many developing and emerging countries, with often deficient or missing pesticide related regulative systems, legislation and education [15]. Typical examples are found in the production of rice and cashew in Southeast Asia and tobacco, bananas, pineapple and coffee in Africa, Central America and the Caribics [15].

Field studies worldwide showed that misuse of pesticides in various sectors of agriculture is often associated with health problems and environmental contamination [16,17,18,19,20].

Epidemiological evidence in this field is still limited, and there is no standardized case definition for acute pesticide poisoning. However, the WHO released a bulletin in 2008 which provides a comprehensive list showing the likelihood of specific acute symptoms to occur in association with specific groups of pesticides and a classification of symptoms for low, high and moderate poisonings [21].

According to a report by the European Commission [22], almost 6000 tons of pesticides (5,811,711 kg) were imported to the Dominican Republic (D.R.) in 2010, and more than 50% of these substances are not authorized in the EU. Paraquat and malathion were imported to the D.R. in 2010 in quantities of 730,000 L and 2000 kg of commercial product, respectively.

Acute effects of pesticide use may represent a significant health risk for the population of coffee-producing countries [23,24]. Data from Central America with an agrarian-ecological situation similar to D.R. show an incident rate of acute pesticide poisoning of 35 per 100,000 in the general population [25]. Corriols et al. [26] reported for Nicaragua an incidence rate of 2.3% for self-reported acute pesticide poisonings, 90% of them related to occupational exposure. These findings underline the necessity to obtain reliable and sound information about pesticide exposure and associated acute symptoms in other countries that might face high incident rates, like D.R. One of our principle objectives was to create a (low cost) approach for field studies even feasible under different conditions e.g., concerning poor infrastructure. However, the study design should generate scientifically sound results.

To the best of our knowledge, our investigation is the first to demonstrate and quantify the impact of pesticide use in the D.R. In a companion article in the special issue [12], we report about cytological findings (genotoxicity, cytotoxicity) in exfoliated buccal cells of coffee farmers in the Jaraboacoa region. In the context of the present study, we test the hypothesis that exposure to pesticides as involved in conventional coffee cultivation is associated with a higher frequency of acute or subacute symptoms than reported by organic farm workers.

## 2. Materials and Methods

### 2.1. Participants

**Figure 1:**
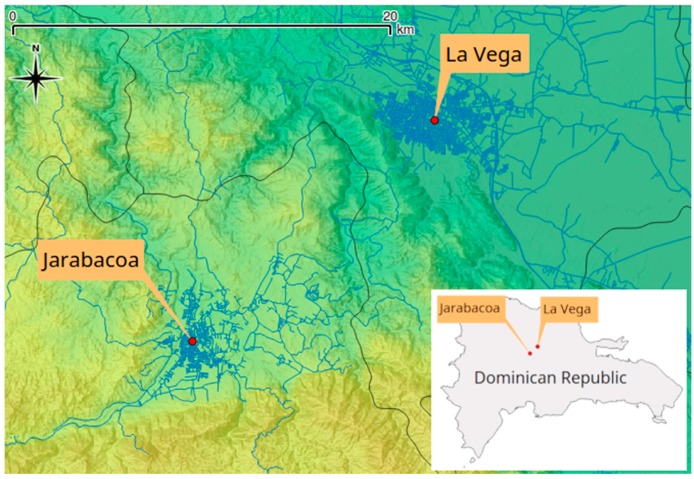
Location of the study area of Jarabacoa, province La Vega, D.R. Cartography licensed under CC BY-SA 2.0, © OpenStreetMap contributors (www.openstreetmap.org).

The Jarabacoa coffee region (La Vega, D.R.), located in the subtropical Central Mountain Range, was selected for the field study, as it is well known especially for conventional and organic agriculture (Figure 1).

The survey primarily contained a structured interview as well as Buccal Micronucleus Cytome Assay (BMCA) in an associated study [12]. Exposed pesticide farmers and non-exposed farmers were recruited through snowball sampling. Initially, workers, who were recruited by our local co-workers, were asked to nominate other male participants of their village, who meet eligibility criteria. The inclusion criteria were: (1) pesticide use for greater than five years, (2) pesticide use at least three weeks before investigation, and an (3) age greater than 18 years.

The control group were also farm workers of the same region but practicing organic farming for at least five years.

Informed consent was obtained from each individual by the trained interviewers. Data about demographics and exposure variables were collected by an adapted questionnaire (see below).

More details on recruitment of participants, and their demographic and socioeconomic status are described in our previous paper [12]. The study was approved by Centro Medico, Puerto Plata, D.R. (V02/15).

### 2.2. Questionnaire

The survey was based on standardized questionnaires supplemented by some symptoms communicated by local health authorities. The forms for the exposed group consisted of 39 questions (122 response options resp. items), and those for the control group included 27 questions (89 items). Questions were of two forms: (1) the yes/no question, which offers a dichotomous choice, and (2) the multiple choice question, which offers several fixed alternatives.

The data collected included socio-demographic features, symptoms (acute/subacute health problems) and indicators of exposure such as working conditions (pesticides applied, safety measures, cleaning procedures, etc.), knowledge and attitudes towards pesticides as well as the housing situation (proximity to cultivation area, handling of pesticides leftovers, etc.).

Before starting the investigation, several pilot interviews were carried out with farm workers (not included in the sample), in order to adjust the questionnaire accordingly. Data were collected using face-to-face interviews conducted by interviewers from the study areas. The interviewers were specifically trained for the project by a half-day workshop of the research team.

The questionnaire included questions related to health symptoms and covered the occurrence of 19 different symptoms in the last six months, which can be indicative of possible toxic effects by pesticides. Two categories of effects can be distinguished: (1) local irritation symptoms and (2) systemic effects. The following symptoms were included: headache, vision problems, dizziness, strong fatigue, exhaustion, sleeplessness, nausea/vomiting, stomach pain, diarrhoea, excessive salivation, burning eyes, skin irritations, skin rashes, runny nose, watering eyes, breathing difficulties, cough, irregular heartbeat, and twitching/trembling.

### 2.3. Statistical Analyses

The dependent variables of interest were the symptoms that subjects reported having occurred during the last six months. These symptoms were assessed as dichotomous variables. The independent variables were working with pesticides vs. organic farming (1/0 coded), age and body mass index (BMI) as continuous variables, and education as a categorical variable (0 none, 1 compulsory, 2 secondary).

Sample size was determined for the assessment of buccal cell anomalies (companion paper). We conducted a power analysis based on the requirement that an odds-ratio of 2 should be detected if the prevalence in the control group is 25% and the significance level is 5% (two-sided). Under these conditions, the study has a power to detect this effect of 81%.

Features of groups were compared by Mann—Whitney tests, chi-square tests or Fisher’s exact probability tests, as appropriate. No correction for multiple endpoints was applied. The presence of symptoms was analyzed by unconditional logistic regression with pesticide exposure as independent variable and age, education and body mass index as covariates. Analyses were done by Stata 13.1 (StataCorp, College Station, TX, USA).

*p* values < 0.05 were considered to be statistically significant.

## 3. Results

Thirty-eight farm workers exposed to pesticides and 33 non-exposed workers, in total 71 persons, were surveyed. In the pesticide exposed group, the mean age was 34.6 years and in the non-exposed group 48.5 years. Both groups, organic farmers and pesticide users, had similar education, lifestyle and dietary habits (tobacco smoking and chewing). However, we found some significant differences (Table 1). Organic farmers were older, had higher BMI and considered pesticides more often harmful.

Pesticides were prepared, mixed and applied by the workers who were also responsible for the disposal of containers. The consequent usage of masks and gloves (all the time) was only reported by 4–5% of the exposed group, increasing to just 11–13% when considering workers who reported using masks and gloves at least half of the time. While hand washing immediately after spaying at the site or at home was performed always by a relatively high percentage of the workers (68%, and 74%, resp.), clothes were not changed after spraying by 55%. Sixty-one percent of the respondents are disposing left over pesticides in their yard. Further important characteristics of pesticide usage are shown in Table 2.

Only one participant was unable to provide information about the types of pesticides used. The majority of pesticides reported by the other 37 farm workers were herbicides, followed by fungicides and insecticides.

On average, pesticide workers were using five different chemicals (minimum 2; maximum 10). The majority of the reported herbicides were organophosphates with glyphosate as the most common substance. The use of paraquat was reported by 38 workers, while only a few of them mentioned the use of malathion, one participant reported 2,4-D (2,4-dichlorophenoxyacetic acid). Among the insecticides, synthetic pyrethroids (e.g., cypermethrin) and carbamate (e.g., methonyl) were mentioned most often.

The results of our analysis, taking age, education level, and body mass index into account, are shown in Table 3 reporting the prevalence/frequency of each symptom and the odds-ratio for pesticide exposure. Almost all types of symptoms covered by the questionnaire (except headache and cough) were more frequently reported by exposed workers.

In nine out of 19 symptoms reported by the participants, the difference between pesticide workers and organic farmers reached significance. Skin rashes were reported by 29% of the exposed workers but by none of the unexposed workers. For all other symptoms, the relative risk ranged from 0.84 (cough, n.s.) up to 15.1 (excessive salivation, *p* < 0.001).

## 4. Discussion

A large variety of different pesticides is used in coffee plantations. A recent study identified 117 different pesticides in unwashed raw coffee imported to Europe [27]. The list of applied pesticides may include insecticides (often applied to fight the coffee berry borer, *Hypothenemus hampei*), fungicides and herbicides that are often used in combination.

Applied substance classes include, among others, organophosphates, carbamates, organochlorines, and pyrethroides. Due to this high chemical diversity, (sub)acute symptoms of exposed plantation workers may vary greatly depending on the substances actually used.

Our intention was to assess the feasibility of different epidemiological methods (including buccal cells micronucleus test [12]) for field studies under challenging local conditions, and to investigate whether application of pesticides is associated with (sub)acute and chronic effects on well-being and health.

In total, both groups reported a considerable number of symptoms. However, we found striking differences in the prevalence between the exposed workers and the non-exposed group. Generally speaking, mostly irritative (e.g., runny nose, watering eyes, skin rashes), intestinal and systemic symptoms (e.g., dizziness, irregular heartbeat) are reported to a much higher degree by the exposed workers. Some symptoms, which may be related to the parasympathic nervous system (e.g., excessive salivation), were seen significantly more frequently in the exposed group. This finding is plausible considering the neurotoxic properties of most of the pesticides reported by the workers (e.g., organophosphates). The effects could be interpreted as a sign of neuro-vegetative disturbance e.g., by acetylcholine esterase inhibitors [28]. These observations are in line with the list of pesticides reported by the workers.

Different studies demonstrated extensive short-term health effects in farmers exposed to pesticides similar to our study [29]. A similar set of acute effects was found in a study in small scale coffee farmers in Tanzania, with skin and eye irritations, flu-like signs, and headache as most prevalent symptoms [30]. A cross-sectional study with 6222 agricultural workers was investigated by questionnaire and blood-tests (serum acetylcholinesterase, AChE) in Thailand [31]. Most frequent self-reported symptoms were coughing, being tired, dizziness, and dry skin and irritation. The main symptom reported by participants with abnormal serum AChE levels was dizziness. These results are quite in line with our findings considering that organophosphates were widely used by the pesticide workers.

Acute symptomatic effects of organophosphates have also been described for coffee-workers in Tanzania, which showed a high incidence of cough, headache, feeling weak and dizziness during spraying periods [32]. Even though we found differences in breathing difficulty, going along with several other reports considering pesticides and respiratory health [33,34,35,36], we did not find a difference in reported cough. One possible reason is that our exposed group was significantly younger, and research about self-reported health shows that older subjects rate their health poorer than younger ones [37,38,39], probably representing indeed a poorer health status [37]. 

It has to be mentioned that, due to inappropriate handling of left-overs or clothes, farm workers, their families, and also even the communities might undergo exposures. Such poor handling relates to lack of awareness of the toxic potential of pesticides. Although almost two-thirds of our respondents think that pesticides are harmful for health, they are acting careless in terms of disposal or other precautionary measures to avoid contamination of the environment and/or themselves (e.g., glove or mask usage) and their family.

It must be noted that the majority of sprayers reported using paraquat and malathion. Malathion is classified—like glyphosate—as probably carcinogenic by the International Agency for Research on Cancer (IARC) [40,41]. Paraquat, which is banned in Europe, is known to pose a health risk to the workers as well as to the public due to persistent use in developing countries [42]. 

Overall, inappropriate use of toxic pesticides combined with a weak or more or less absent legislative framework as well as missing inspection by authorities are some major reasons for the higher prevalence of pesticide related symptoms and diseases in developing countries. Low education of the rural population, lack of information and training on pesticide safety, poor spraying technology, and inadequate personal protection are key elements of higher exposure in field workers (e.g., [43,44,45]). In general, knowledge of the main determinants of pesticide exposure in countries of the global south is often poor. In summary, reduction of pesticides production and application is strongly needed.

### Limitations and Recommendations for Future Research

The present sample size results from our priority aim to investigate chronic health effects of pesticide exposed workers by buccal micronucleus cytome assay (BMCA) in the accompanying study [12]. However, our sample size determination ensured sufficient power for symptoms analysis as well.

Symptoms were reported by participants of a study with its main focus on objective measures of pesticide induced cytotoxicity and genotoxicity. Respondents were aware of the aim of the study and of their exposure status. Thus, some reporting bias cannot be excluded as more than half of the exposed participants rated pesticides as a hazard to their health.

Another bias might result from the difference in age between exposed and non-exposed group. Although age was included as potential confounder, residual confounding cannot be excluded.

Many investigations faced difficulties to describe the exposure of the workers to specific pesticides more precisely. It is often attributed to organizational difficulties in the field and/or insufficient financial support (high laboratory costs) to identify pesticides by environmental monitoring (soil, water) at the site and/or by human biomonitoring (blood, urine). We could not entirely determine which specific pesticide is responsible for our findings due to the mostly combined exposure to many different substances. Previous research has shown that, in more than one third of the studies, interaction effects between different pesticides exist, mainly leading to synergic effects [46], so that regulatory standards for pesticide mixtures are recommended [47]. The relationship between amount of pesticide exposure and effects remains unclear because exposure intensity could not accurately be assessed in this study. It also remains in the dark which exposition routes contribute in the given sample, since human pesticide exposure generally happens via various routes (e.g., via drinking water [48], food [49], direct contact with soil or soil inhalation [50], with drinking water and food representing even more severe health risk factors than soil [51]), but whatever the route we have no reason to assume that our exposed and non-exposed group differ systematically in other than occupational-caused uptake.

Workers are exposed to complex mixtures of numerous chemicals, rather than to individual chemicals. Additionally, different uptake routes have to be taken into account. Future studies should refer more in detail to the complex situation of pesticide application and health effects. Therefore, specific methods might be applied to identify the internal burden of pesticides in farm workers. The exposure assessment involves additionally the environmental concentrations of pesticide residues, inter alia in water and soil. However, it should be noted that such approaches using human biomonitoring and environmental monitoring methods are definitely of much higher organizational and financial dimensions.

However, even just focusing on subjective symptoms, our results show the need for restrictive pesticide policies and regulations. Generally, our results are in line with other studies in pesticide users, so spuriously increased risks are unlikely.

## 5. Conclusions

In our field study that was mainly conducted to investigate specific cytotoxic effects of pesticides, further insight into (sub)acute health effects of pesticide exposure in conventional coffee production was obtained by inquiry into symptoms experienced by the farmers. Our findings support adverse effects of unprotected occupational pesticide exposure on subjective wellbeing and health.

Taking these findings and previously reported effects on integrity of buccal cells together [12], we can conclude that pesticide exposed workers not only suffer from impaired well-being and impaired recovery during leisure time due to the symptoms, but are also at an increased risk of developing cancer.

Our study shows that conventional farming under the current socio-economic situation in the studied area may be associated with serious adverse health effects in pesticide exposed workers. We suggest more support for organic farming by regulatory measures as well as structural aid as a countermeasure. A more restrictive pesticide policy and a stronger framework of regulations and control for worker protection are equally advised.

## Figures and Tables

**Table 1:** Comparison of personal lifestyle and educational background for non-exposed and pesticide exposed male farm workers in Jarabacoa, mean +/− SD and percent.

Attributes	Non-Exposed (*n* = 38)	Pesticide Exposed (*n* = 33)	*p*-Value
Age (years)	48.5 ± 20.7	34.6 ± 14.1	0.004
Body mass index (BMI) (kg/m²)	24.9 ± 3.1	23.4 ± 2.8	0.046
Education			0.228
None	5 (15%)	3 (8%)	
Compulsory	17 (52%)	27 (71%)	
Secondary	11 (33%)	8 (21%)	
Mother Farmer	22 (67%)	30 (79%)	0.289
Father Farmer	30 (91%)	37 (97%)	0.331
Tobacco chewing	4 (12%)	9 (24%)	0.237
Smoking	3 (9%)	2 (5%)	0.658
Eating fish (days/week)	0.3 ± 1.2	0.2 ± 0.4	0.946
Eating meat (days/week)	2.6 ± 1.7	2.3 ± 1.9	0.048
Pesticides harmful for health according to the respondents	30 (91%)	24 (63%)	0.011

**Table 2:** Characteristics of pesticide use in pesticide exposed farmers.

Characteristics	Unit/Category	Mean ± SD or n (%)
Pesticide spraying	years	18.3 ± 11.7
Spraying	days/week	1.5 ± 1.5
Last spraying	days ago	10.2 ± 6.1
Wearing gloves	always or half of the time	4 (11%)
Wearing masks	always or half of the time	5 (13%)
Storage of equipment	inside of the home	4 (11%)
Where equipment is cleaned	nearby river or creek	4 (11%)
	outside the yard	31 (82%)
	at home	2 (5%)
Washing hands at spraying site	always	26 (68%)
Washing hands immediately at home	always	28 (74%)
Washing hands later at home	always	7 (18%)
Washings hands before going to bed	always	21 (55%)
Changing clothes after spraying	no	17 (45%)
Where left overs of containers disposed	in solid waste disposal	2 (5%)
	in yard	23 (61%)
	save it for next time	10 (27%)
	keep it at the house	2 (5%)
	keep it in the spaying device for the next time	1 (3%)
Container further used	yes	5 (13%)

**Table 3:** Symptoms reported by non-exposed and pesticide exposed workers. Odds ratios (OR), 95% confidence intervals and *p*-values for pesticide use corrected for age, body mass index and education (sig. results in **bold**).

Symptom	Non-Exposed *n* = 33	Pesticide Exposed *n* = 38	OR (95% CI)	*p*-Value
Headache	78%	77%	0.97 (0.28–3.35)	0.956
Visual problems	36%	62%	2.93 (0.79–10.88)	0.108
**Dizziness**	**14%**	**57%**	**8.19 (2.01–33.31)**	**0.003**
Nausea/vomiting	28%	47%	2.23 (0.73–6.79)	0.159
**Excessive salivation**	**8%**	**58%**	**15.14 (3.08–74.47)**	**<0.001**
**Strong tiredness**	**39%**	**76%**	**4.91 (1.51–15.93)**	**0.008**
Exhaustion	29%	55%	3.01 (0.96–9.39)	0.058
**Stomach ache**	**25%**	**71%**	**7.38 (2.13–25.57)**	**0.002**
Diarrhea	15%	24%	1.74 (0.45–6.68)	0.421
Sleeplessness	58%	75%	2.12 (0.67–6.76)	0.203
Burning eyes	43%	51%	1.39 (0.47–4.16)	0.551
Skin irritations	10%	25%	2.85 (0.65–12.60)	0.166
**Runny nose**	**52%**	**78%**	**3.18 (1.01–10.05)**	**0.048**
**Difficulty breathing**	**26%**	**60%**	**4.33 (1.32–14.22)**	**0.016**
**Irregular heartbeat**	**24%**	**65%**	**6.05 (1.72–21.25)**	**0.005**
**Watering eyes**	**37%**	**66%**	**3.26 (1.04–10.27)**	**0.043**
**Skin rashes**	**0%**	**29%**	**-**	**0.001**
Cough	49%	45%	0.84 (0.29–2.43)	0.747
Twitching/tremor	10%	26%	3.19 (0.70–14.56)	0.135
